# Aggressiveness in systemic anticancer therapy at the end of life in an oncology center

**DOI:** 10.1097/j.pbj.0000000000000248

**Published:** 2024-03-08

**Authors:** José António Ferraz-Gonçalves, Inês Silva, Patrícia Redondo, Michael Sapateiro Luís

**Affiliations:** a Instituto Português de Oncologia, Porto—Palliative Care Service, Porto, Portugal; b Instituto Português de Oncologia, Porto—Outcomes Research Lab, Porto, Portugal

**Keywords:** Cancer treatment, treatment aggressiveness, end of life, chemotherapy at the end of life, systemic anticancer therapy

## Abstract

**Introduction:**

An increasing aggressiveness in cancer treatment at the end of life (EoL) has been reported in several, but not all, countries. This study aimed to see how aggressive cancer treatment is at the EoL in an oncology center.

**Methods:**

Retrospective study of patients 18 years or older with a solid cancer diagnosis who died in 2017. The focus was systemic anticancer therapy (SACT), excluding hormonotherapy.

**Results:**

In 2017, 2024 patients with solid tumors died. Of those patients, 1262 (62%) were male, and the median age was 69 (range 19–97) years. The most frequent primary cancer was lung cancer, followed by colorectal and stomach cancers, and 740 (37%) patients had metastatic disease. The median interval between SACT and death was 61 days. Of the patients undergoing SACT, 216 (27%) did it in the last month of life, 174 (22%) between 8 and 30 days from death, and 42 (5%) in the last week. On multivariable analysis, head and neck, colorectal, breast, and melanoma primaries; age group (older than 65 years); and metastatic disease had statistical significance associated with SACT. Of these variables, only metastatic disease is more likely to undergo SACT.

**Conclusion:**

This study confirms the relatively frequent aggressiveness in cancer treatment at the EoL. Taking into consideration previously published data, it can be tentatively concluded that the use of SACT increased in the last month and the last week of life.

## Introduction

Cancer is one of the leading causes of death in developed countries. Cancer is now the second cause of death in Portugal, following cardiovascular diseases.^1^ In contrast to cardiovascular disease, the percentage of which decreases, the cancer death rate increases. For example, the rates of cardiovascular and cancer deaths were 38.7% and 20.3%, respectively, in 2000, and in 2019, they were 29.9% and 25.5%.^1^ The increasing average life expectancy is undoubtedly at least a part of the explanation for that change.

Cancer treatment is improving continuously, but most patients still reach a far advanced phase, where the treatment objective must change from prolonging life to quality of life and comfort. However, sometimes, the anticancer treatment is prolonged too far, negatively affecting the well-being of patients and families. In the first decade of the millennium, Earle et al^2^ published studies on the problem of the aggressiveness of cancer therapy at the end of life (EoL), detecting an increase in the use of chemotherapy in the last weeks of life. Earle et al^3^ tried to establish criteria for the quality of cancer care at the EoL. The criteria address various aspects, and those concerning chemotherapy included the institution of new anticancer therapies or continuing treatments very near death.^3^

The attitude toward aggressivity and its trend at the EoL varies among countries.^4-7^ A nationwide study recently published in Portugal about the aggressiveness of care at the EoL in patients with cancer dying in hospital, including 92,155 patients, showed that 7% of them underwent chemotherapy.^8^

We studied patients followed at an oncology center with solid tumors who died in 2002 to find how close-to-death chemotherapy was used in our oncology center.^9^ Of the patients who underwent chemotherapy, 3% and 13% were on it in the last week and the last month, respectively. Since 2002, much improvement has been occurring in cancer treatment, mainly in systemic drugs, not only chemotherapy but also with the introduction of other systemic therapies such as immunotherapy and targeted therapy. The main objective of this study was to evaluate the use of systemic anticancer treatment (SACT)—immunotherapy, chemotherapy, and targeted therapy—at the EoL in contemporary conditions.

## Methods

This is a retrospective cohort study regarding the EoL of adults who died in 2017 in a Portuguese Comprehensive Cancer Center (Portuguese Institute of Oncology of Porto—IPO Porto). Patients' inclusion criteria include date of death in 2017 documented on the administrative records of IPO Porto, age 18 years or older in 2017, and diagnosis of solid cancer recorded in the IPO Porto Diagnosis Registry, using the International Classification of Diseases for Oncology, third edition.

Patients' exclusion criteria include benign neoplasms and in situ, skin tumor nonmelanoma, hematological cancer; and patients with two or more diagnoses of cancer in the registry database.

As the focus of this study was the use of SACT at the EoL, aggressiveness was defined as the use of SACT in the past 30 days of life, like the definition of Earle et al^2^ “the last dose of chemotherapy within 14 days of death, a new chemotherapy regimen starting less than 30 days before death, but not including the other elements used in that definition.

Demographic and clinical data, including age, sex, and date of death, were abstracted from clinical and administrative records. Cancer-related data included the date and cancer diagnosis, documentation of metastatic disease, and death at IPO Porto. Cancers with a frequency ≤2% were classified as others. SACT was considered only immunotherapy, chemotherapy, and targeted therapy. The date of last treatment before death was stratified by intervals of 1–7 days, 8–30 days, 31–90 days, 91–180 days, and more than 180 days from the last treatment until death. Only the contact with IPO Porto Palliative Care Service was considered palliative care. The information regarding patients who did not have any SACT was also evaluated.

The institutional ethics committee approved this study.

### Statistical analysis

Descriptive statistics for categorical data were summarized as proportions with 95% confidence intervals (95% CI), and continuous variables were summarized as median and range.

The study population was described, followed by a comparative analysis through logistic regression. We performed a univariate analysis considering lung cancer as the reference since it has the highest incidence in our population. The multivariate analysis was performed using logistic regression, with the variables statistically significant in the univariate analysis. The significant level considered was *P* < .05. We considered at least 15 patients for any variable to perform statistics tests.

Finally, all analyses were based on complete cases using R Studio.

## Results

In 2017, 2024 patients with solid tumors followed in the IPO died. Of those patients, 1262 (62%) were male, and the median age was 69 (range 19–97) years. The most frequent primary cancer was lung cancer, followed by colorectal and stomach cancers, and 740 (37%) patients had metastatic disease (Table 1). The median interval between SACT and death was 61 days. Of the patients undergoing SACT, 216 (27%) did it in the last month of life, 174 (22%) between 8 and 30 days from death, and 42 (5%) in the last week (Table 2). Chemotherapy was, by far, the most frequent SACT.

**Table 1.** Demographic and clinical data

	2024 patients
Age, years, median (range)	69 (19–97)
Age groups, years, n (%)	
18–34	21 (1)
35–44	63 (3)
45–54	218 (11)
55–64	433 (21)
65–74	584 (29)
>74	705 (35)
Age groups, years, (2 groups), n (%)	
<65	735 (36)
≥65	1289 (64)
Sex, n (%)	
Male	1262 (62)
Female	762 (38)
Primary cancer, n (%)	
Lung	379 (19)
Colorectal	251 (12)
Stomach	248 (12)
Breast	203 (10)
Head—neck	201 (10)
Prostate	150 (7)
Gynecological	100 (5)
Sarcoma	63 (3)
Melanoma	61 (3)
Skin other	57 (3)
Pancreas	55 (3)
Esophagus	48 (2)
Bladder	41 (2)
Other	167 (8)
Metastases, n (%)	
Yes	740 (37)
No	1284 (63)

n, number.

**Table 2.** Time from the last SACT until death

	n (% of total)	% of SACT
SACT—total		
Patients—total	796 (39)	100
Patients in last month of life	216 (11)	27
“1–7” (days)	42 (2)	5
“8–30” (days)	174 (9)	22
“31–90” (days)	296 (15)	37
“91–180” (days)	145 (7)	18
>180 (days)	139 (7)	17
Chemotherapy	691 (34)	87
Targeted therapy	90 (4)	11
Immunotherapy	15 (1)	2

n, number; SACT = systemic anticancer therapy.

On univariable analysis, the statistically significant results with a higher probability of undergoing SACT were patients younger than 65 years and with metastatic disease. The patients with head and neck, colorectal, stomach, breast, melanoma, and other primaries had a statistically significantly lower probability of undergoing SACT relative to lung primary (Table 3).

**Table 3.** Differences between patients who did and did not undergo SACT by univariable analysis

	SACT			
	Yes (n= 771), n (%)	No (n= 1005), n (%)	*P*	OR
Age group (years)				
<65	414 (54)	297 (30)		
≥65	357 (46)	708 (70)	**<0.001**	0.30
Sex				
Female	283 (37)	440 (44)		
Male	488 (63)	565 (56)	0.2	1.13
Primary cancer type				
Lung	201 (26)	178 (18)		
Head—neck	84 (11)	117 (12)	**0.01**	0.64
Colorectal	102 (13)	149 (15)	**0.002**	0.61
Esophagus	23 (3)	25 (2)	0.504	0.81
Stomach	110 (14)	138 (14)	**0.034**	0.71
Gynecological	49 (6)	51 (5)	0.473	0.85
Breast	65 (8)	138 (14)	**<0.001**	0.42
Melanoma	18 (2)	43 (4)	**<0.001**	0.37
Pancreas	50 (7)	117 (12)	0.644	1.14
Sarcoma	31 (4)	24 (2)	0.284	1.35
Other	38 (5)	25 (2)	**<0.001**	0.38
Metastases at diagnosis				
Yes	367 (48)	337 (34)		
No	404 (52)	668 (66)	**<0.001**	2.09

Bladder, prostate, and skin cancers were withdrawn from the analysis because the number of patients was <15. Therefore, the total number considered for this analysis was 1776.

Bold—statistical significance.

n, number; OR, odds ratio; *P*, probability; SACT, systemic anticancer therapy.

On multivariable analysis, head and neck, colorectal, breast, and melanoma primaries; age group (older than 65 years); and metastatic disease had statistical significance associated with SACT. Of these variables, only metastatic disease is more likely to undergo SACT (Table 4).

**Table 4.** Multivariable logistic regression

	OR	CI (95%)		*P*
		Lower	Upper	
Lung	1			
Head—neck	0.61	0.42	0.89	**<0.001**
Colorectal	0.70	0.50	0.98	**0.041**
Stomach	0.74	0.53	1.03	0.07
Breast	0.42	0.29	0.62	**<0.001**
Melanoma	0.43	0.23	0.79	**0.008**
Age group (≥65)	0.35	0.28	0.42	**<0.001**
Metastases (yes)	1.40	1.13	1.74	**0.002**

Bold—statistical significance.

CI, confidence interval; OR, odds ratio; *P*, probability.

Of the 2024 patients, 978 (48%) died in the IPO wards, 556 (57%) of them in the palliative care department.

## Discussion

In this study, the percentage of patients who underwent SACT in 2017 was 39%. Patients with metastatic cancer and the younger ones had a higher probability of being submitted to chemotherapy. Of the patients who underwent SACT, 27% did it in the past 30 days of life and 5% in the last week. SACT so close to the EoL is usually inappropriate and indicates treatment aggressiveness.

The aggressiveness of cancer treatments near the EoL is widespread. Earle et al^2^ first published a study indicating that the treatment of patients with advanced cancer was becoming increasingly aggressive, and that aggressiveness continued later in life in the United States. Several reasons were added for recommending treatments with minimal potential benefits^10^: providing hope, avoiding difficult discussions about changing the focus of treatment from fighting cancer to providing symptomatic and supportive care, and financial incentives. Patients may also want to proceed with or start new treatments because they do not understand their actual prognosis, have unrealistic expectations about the benefits of chemotherapy, want to be a "fighter," or feel that doing anything is better than doing nothing.^10^ Since the first report on the aggressiveness of chemotherapy and other treatments, many other studies have been conducted in several countries. For example, in Ontario, Canada, between 1993 and 2004, with each successive year, patients had a 1% increased risk of encountering some aggressive intervention at the EoL,^4^ however, not as much as in the United States. The authors added as one possible explanation for the difference between Canada and the United States concerning aggressiveness that the financial incentives in the United States may induce some practitioners to continue providing anticancer treatment. This possibility is supported by a French study that found that patients who died in private for-profit facilities were significantly more likely to receive chemotherapy in the final month than patients who died in public hospitals.^6^

The treatment aggressiveness is not high or increasing universally. There are examples of low levels of aggressiveness in Austria^11^ and even decreasing aggressiveness, although still at a high level.^7^

It may be argued that the introduction of immunotherapy and targeted therapy allowed treating patients less aggressively and prolonged survival. However, when used in the last weeks of life, in patients who are deteriorating despite their use, they are inadequate and may be classified as aggressive treatment.

In this study, 57% of the patients' deaths in the hospital occurred in the palliative care department. Palliative care and oncology are not opposed fields; they should be integrated whenever possible. Palliative care can minimize the treatment aggressiveness at the EoL. However, patients are often referred too late, as many patients receive palliative care only in the last three days of life^10^ when earlier hospice enrollment is one factor associated with perceptions of better EoL care by family members.^12^ The effect of palliative care on the treatment aggressiveness is differently reported. In one study, timely consults attenuated aggressiveness.^13^ On the contrary, in a nationwide Portuguese study, there was no association between hospital palliative care services at the hospital of death and the aggressiveness of cancer care.^8^

In a study in our hospital in 2002,^9^ 13% of the patients who underwent chemotherapy did it in the last month of life and 1% in the last week. In this study, done 15 years later, the rate of patients submitted to aggressive systemic therapy near the EoL roughly doubled. Nevertheless, those data, mainly last week's, should be interpreted cautiously because the numbers are low and so susceptible to confounding factors.

This study has some weaknesses. It was performed in only one hospital, which was a specialized oncology center. Therefore, the data cannot be generalized as the oncology practice in Portugal. The aggressiveness of treatment is not limited to SACT. Several other interventions can contribute to the aggressiveness of cancer care at the EoL. The other aspects of the treatment aggressiveness will be addressed in another study.

## Conclusion

Cancer treatment aggressiveness seems relatively frequent at the EoL, as the data of this study on the use of SACT confirm. Compared with previously published data, it can be tentatively concluded that there is an increased use of SACT in the last month and the last week of life in this oncology center.

## Ethical standards statement

This study was approved by the institutional ethics committee.

## Conflict of interest

The authors declare no conflicts of interest.
